# The efficacy and safety of complementary and alternative medicine in the treatment of nausea and vomiting during pregnancy: A systematic review and meta-analysis

**DOI:** 10.3389/fpubh.2023.1108756

**Published:** 2023-03-09

**Authors:** Mo-Yao Tan, Shi-Hong Shu, Run-Lei Liu, Qian Zhao

**Affiliations:** ^1^Chengdu Integrated TCM and Western Medicine Hospital, Chengdu, China; ^2^Clinical Medical School, Chengdu University of Traditional Chinese Medicine, Chengdu, Sichuan, China; ^3^School of Pharmacy, Chengdu University of Traditional Chinese Medicine, Chengdu, Sichuan, China; ^4^School of Modern Chinese Medicine Industry, Chengdu University of Traditional Chinese Medicine, Chengdu, Sichuan, China

**Keywords:** complementary and alternative medicine, nausea and vomiting, meta-analysis, pregnancy, GRADE

## Abstract

**Background:**

Complementary and alternative medicine (CAM) therapies are widely used for nausea and vomiting during pregnancy (NVP) due to the limitations of conventional medicine. However, their efficacy and safety remain controversial. Therefore, this meta-analysis was performed to assess the improvement of CAM therapy on NVP.

**Methods:**

Randomized controlled trials (RCTs) were searched for where the trial group was CAM and the control group was a conventional medicine or a placebo for NVP. This was done *via* 8 databases, including PubMed, EMBASE, the Cochrane Library, Web of Science, China National Knowledge Infrastructure, Wanfang, SinoMed, and VIP, from inception to October 25, 2022. The Grades of Recommendation, Assessment, Development and Evaluation (GRADE) was used to assess the quality of evidence. The Stata 15.0 software was used to perform the meta-analysis.

**Results:**

Thirty-three RCTs were included in this study. The acupuncture treatment was superior to conventional medicine at the effective rate [RR = 1.71, 95% CI (1.02, 2.86), *P* = 0.042; Low-quality evidence]. Ginger had more significant effects than conventional medicine at the Rhodes index [WMD = −0.52, 95% CI (−0.79, −0.24), *P* ≤ 0.001; Moderate-quality evidence] and it had the same effect as drugs to relieve vomiting [SMD = 0.30, 95% CI (−0.12, 0.73), *P* = 0.160; Low-quality evidence]. Compared with placebo, ginger had a higher effective rate [RR = 1.68, 95% CI (1.09, 2.57), *P* = 0.018; Low-quality evidence], and lower Visual analog scale (VAS) of Nausea [WMD = −1.21, 95% CI (−2.34, −0.08), *P* = 0.036; Low-quality evidence]. Ginger had the same antiemetic effect as placebo [WMD = 0.05, 95% CI (−0.23, 0.32), *P* = 0.743; Low-quality evidence]. Acupressure was superior to conventional medicine at the reduction of antiemetic drugs [SMD = −0.44, 95% CI (−0.77, −0.11), *P* = 0.008; Low-quality evidence], and at the effective rate [RR = 1.55, 95% CI (1.30, 1.86), *P* ≤ 0.001; Low-quality evidence]. Acupressure had the same effect as placebo at the effective rate [RR = 1.25, 95% CI (0.94, 1.65), *P* = 0.124; Low-quality evidence]. Overall, CAM therapy was safer than conventional medicine or a placebo.

**Conclusion:**

The results showed that CAM therapies were able to alleviate NVP. However, due to the low quality of existing RCTs, more RCTs with large sample sizes are needed to validate this conclusion in the future.

## Introduction

Nausea and vomiting are common symptoms of pregnancy, also known as nausea and vomiting during pregnancy (NVP) or morning sickness, as it often occurs in the morning (1). The mechanism is unclear but it may be related to hormonal, immunological, or anatomical changes during pregnancy (2). About 50–80% of pregnant women experience NVP, primarily between weeks 6 and 12 of pregnancy, and subsides by week 20. For 9–20% of women, however, NVP may last longer (2, 3). The symptoms are severe enough to cause fluid and electrolyte imbalance and nutritional deficiencies and require hospitalization (4–6). Thus, NVP significantly impacts the quality of life of pregnant women in terms of social, emotional, and psychological health, as well as increasing the economic burden (7). Although there is medication available to treat NVP, some physicians and patients are reluctant to use these drugs due to reports of fetal malformations associated with antiemetic medications (8). One study showed that many NVP patients did not use drugs, or at least used lower than the prescribed doses, due to a lack of trust in the safety of chemical medications, such as vitamin B6, antihistamines, or H1 receptor antagonists (9).

In recent years, CAM therapy has become common in many western countries, with nearly half of all women of childbearing age using this method (9). Considering that CAM is safe, inexpensive, and convenient, patients and clinicians need to understand and promote CAM for those who do not want to be treated with traditional medications or are dissatisfied with them. CAMs such as ginger, acupuncture, and acupressure, are already widely used in clinical practice. The US Food and Drug Administration (FDA) considers ginger to be a safe herbal preparation (10, 11), containing essential oils that block the vomiting reflex, as well as curcumin and sugorol, which both have anti-nausea, anti-vomiting, sedative, and analgesic effects that may be the key to their action (12). Acupressure and acupuncture have similar mechanisms, by applying pressure and activating mast cells at acupuncture points and specific components such as nerve fibers to release endorphins, thus increasing endogenous antiemetic tension (13, 14).

In the past, there have been some meta-analyses on related aspects, such as that by Kannan Sridharan et al., who conducted a net meta-analysis on interventions for NVP (15). However, they did not compare treatment modalities with first-line drugs, which may be out of clinical reality. Estelle Viljoen and Rebecca conducted meta-analyses of ginger and acupressure for NVP, respectively (16, 17). However, more RCTs have been published to date and a pooled update is urgently needed to ensure the integrity and reliability of the findings.

Due to the controversy surrounding the treatment of NVP with CAM therapy and to update the existing evidence, as well as address the limitations of past meta-analyses, this meta-analysis comprehensively includes updated RCTs of clinically used CAM therapies, including acupuncture, ginger, and acupressure, to explore their differences in efficacy with first-line medications and placebo therapy. This aims to offer clinicians and pregnant women who experience NVP the possibility of an alternative treatment option that is effective, safe, and affordable.

## Methods

This study was registered on PROSPERO (CRD42022375440). It was conducted following the Preferred Reporting Items for Systematic Evaluations and Meta-Analysis (PRISMA) guidelines and the recommendations of the Cochrane Collaboration (18, 19).

### Search strategy

To ensure the completeness and readiness of the literature search, the two authors (MYT and SHS) independently searched eight electronic databases, including PubMed, EMBASE, the Cochrane Library, Web of Science, China National Knowledge Infrastructure, Wanfang Database, SinoMed, and VIP Database, from their inception to October 25, 2022, without age, race, or language restrictions.

The reference lists from published reviews of related directions and the critical articles retrieved were used for further research, as well as the links in the gray literature.

For English databases, a combination of subject terms and free words were used for searching. The English database search included four core components, which used AND operator for logical connections: (1) complementary and alternative medicine (e.g., herb, acupuncture, acupressure, ginger, Shiatsu, Zhi Y, Chih Ya, and Zingiber officinal); (2) pregnancy (e.g., Pregnancies, gestation, and pregnant woman); (3) vomiting (e.g., embese, emesia, and vomitus); (4) nausea.

For the Chinese database, we searched using keywords such as '孕妇,' '妊娠期', 恶心呕吐,' '补充替代疗法', '针灸', '生姜', '指压.'. The detailed retrieval process is shown in Supplementary material 1.

### Inclusion and exclusion criteria

(1) *Type of study*: This meta-analysis only included RCTs on CAM therapy for NVP; case reports, conference papers, and RCTs without relevant outcomes were excluded.
(2) *Type of participants*: As symptoms are judged in NVP, no diagnostic criteria are used. Patients who experienced NVP were included and those who presented with nausea and vomiting in the postpartum period were excluded.
(3) *Type of interventions and controls*: RCTs, where patients in the trial group were treated with CAM therapy, were included, including acupuncture, acupressure, and ginger, while the control group was treated with conventional western medicine or a placebo treatment. If the trial group was treated with a combination of multiple CAM therapies and a control group that was not a conventional medicine or placebo-treated RCT, the literature was excluded.
(4) *Type of outcomes*: Outcome indicators for RCTs included at least one of the following. The primary outcomes were the effective rate, the Rhodes scale, and the number of vomiting. The secondary outcomes were measured using the VAS, dose of antiemetic drugs, and adverse events. VAS allowed participants to score their symptoms using a 10 cm line from 0 (no nausea or vomiting) to 10 (severe nausea or vomiting). The Rhodes index included three nausea items (duration, frequency, pain) and two vomiting items (quantity and frequency), with the five items being scored by the Likert scale. The effective rate was the proportion of the number of people who have significantly improved and recovered symptoms in the number of people in the test or the control group. The effect of CAM was evaluated laterally by reducing the number of vomiting and the reduction of vomiting drugs for patients after using CAM. The adverse events of CAM mentioned in each article were counted and included to evaluate the safety of this measure.

### Screening and data extraction

Two researchers (MYT and RLL) independently read the titles and abstracts for screening based on inclusion and exclusion criteria, and then the selected articles were read in full for final selection. If there was any dispute, it would be resolved by the corresponding author (QZ).

Two researchers (MYT and SHS) independently screened the literature and extracted author, year, region, age, sample, interventions, gestation, acupoint, dose or frequency, duration, and outcome from each RCT. If there was any dispute, it would be resolved by the team discussion. When the data included in RCTs were defective or missing, the first author or corresponding author was contacted to seek data information.

### Assessment of risk of bias

Two authors (MYT and RLL) independently assessed the risk of bias of the included RCTs using the Cochrane Risk of Bias Version 2 (RoB 2) assessment tool (20). RoB 2 has six evaluation items: (1) the random sequence generation, (2) deviations from the intended interventions, (3) missing outcome data, (4) measurement of the outcome, (5) selection of the reported result, (6) and overall bias. Each section is graded “low risk,” “some concern,” and “high risk” according to the actual situation of the article. if there were disagreements, a third author (Qian Zhao) would evaluate until a consensus was reached.

### Grade evaluation

The GRADE approach was used to evaluate the quality of evidence and strength of recommendations (21). GRADE considers the following five elements: the risk of bias, inconsistency, indirectness, inaccuracy, and publication bias. The outcomes of the included literature were classified as very low, low, moderate, and high quality according to the content of the assessment.

### Data analysis

The Stata 15.0 software was used to perform the meta-analysis. The standard mean difference (SMD) or weighted mean difference (WMD) was used for data analysis for continuous variables. The dichotomous variables were analyzed by the risk ratio (RR), with a 95% confidence interval (CI). Due to differences in the study methods, basic characteristics of the participants, and the doses and frequency of the interventions, there was significant clinical heterogeneity in the included studies. So, regardless of statistical heterogeneity, we would use a random effects model to analyze the data. To further analyze and identify sources of heterogeneity, subgroup analyses of sample sizes, types of medications, and year of publication were performed.

To test the robustness of the outcome, a sensitivity analysis was performed by removing included studies one by one or by performing only descriptive analysis. If an article was excluded and the result was reversed, the article would be shown to be a source of heterogeneity and the article was analyzed in depth regarding its sample size and year of publication. Otherwise, the results were robust.

When the number of included articles was more than 10, the underlying publication bias was identified *via* an informal visual examination of a funnel plot. Publication bias was evaluated with Begg's tests (22). When the study provided only the median, the mean, standard deviation, and range were calculated based on the methods described by Hozo et al. (23). *P* < 0.05 was used to indicate statistical significance.

## Results

### Study selection

**Figure 1:**
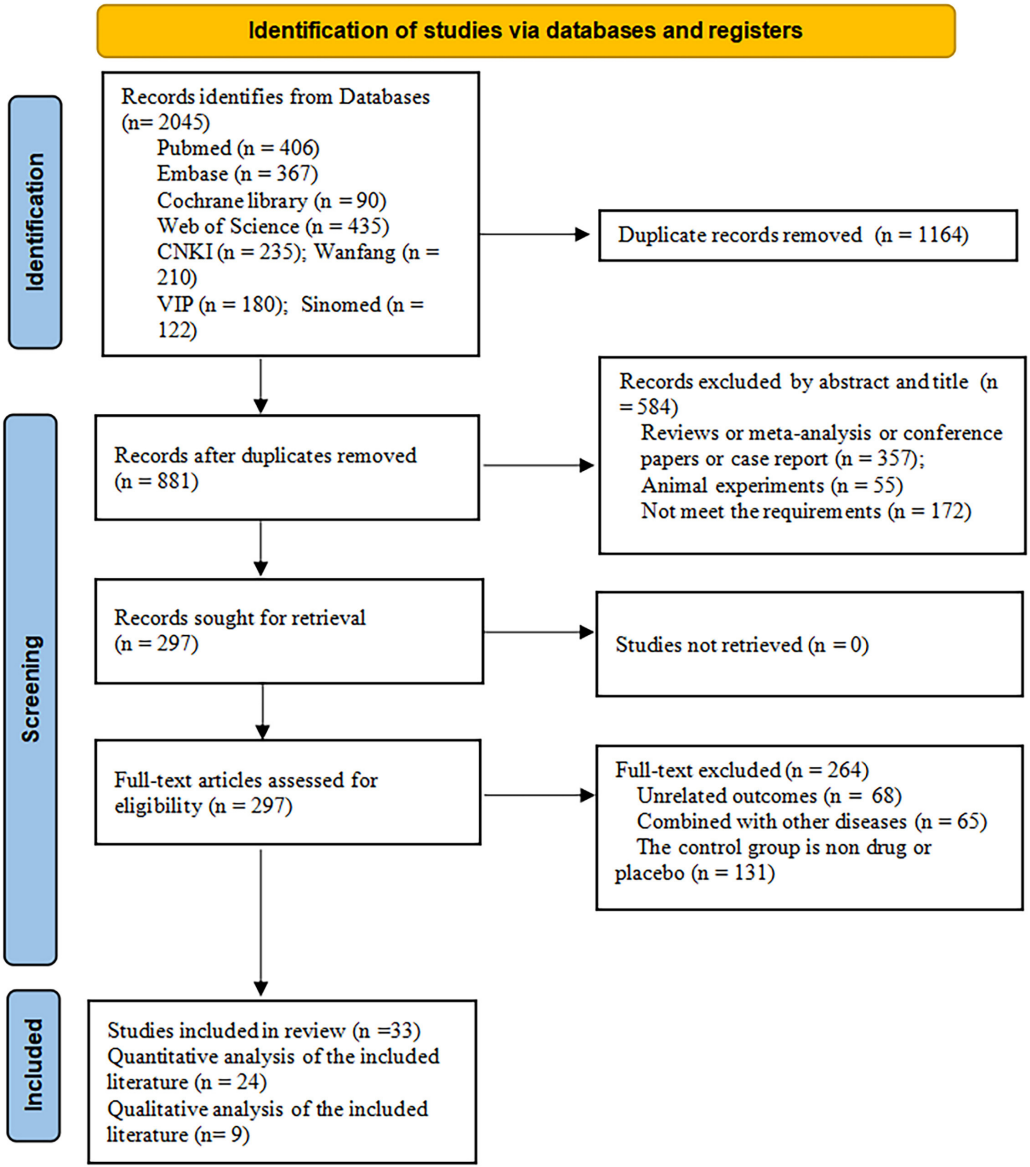
Flow chart of selection studies and specific reasons for exclusion.

A total of 2,045 articles were identified from the eight databases. One thousand one hundred and sixty-four articles were excluded due to duplication. After reading the titles and abstracts, 297 articles remained for full-text review. After scanning the full texts, 33 articles were included. The specific literature screening process is shown in Figure 1.

### Study characteristics

A total of 33 articles (24–56) were included in this paper, of which 24 RCTs (24, 26–30, 33, 35, 37–44, 46, 49–53, 55, 56) were analyzed for quantitative analysis due to sufficient sample size data. The remaining 9 articles (25, 31, 32, 34, 36, 45, 47, 48, 54) were about side effects, for which there were only descriptive discussions provided due to the small sample size data. Three articles (24, 35, 37) only offered the median and interquartile range, which we algorithmically converted to the mean and standard deviation (23).

The included RCTs were published from 2002 to 2022. Eleven (24, 27–29, 31, 33, 35, 36, 38, 39, 45) were conducted in Iran, five (25, 30, 37, 40, 41) in Thailand, five (26, 32, 47, 49, 55) in Australia, four (43, 51–53) in China, two (42, 44) in Malaysia, two (46, 54) in the UK, one (50) in Sweden, one (34) in Indonesia, one (48) in the USA, and one (56) in Croatia. The sample sizes of the included RCTs ranged from 18 to 340. The mean age of pregnant women was between 19 and 35 years old and the gestation period was between 1 and 17 weeks at baseline.

The CAM interventions were classified into acupuncture, acupressure, and ginger. Six articles (49–54) reported on the efficacy of acupuncture with a treatment period of 5 days to 4 weeks. Twelve articles (38–48, 55) reported about acupressure and the treatment period was 1 day to 4 weeks. There were 14 articles (24–37) studying ginger with a dosage of between 500 and 2,500 mg. Also, 1 article (56) had two experimental groups: acupuncture and acupressure, lasting for 1 week.

In the control group, interventions included sodium lactate ringer's injection, dimenhydrinate, vitamin B6, metoclopramide, antiemetics, sodium bicarbonate + phenobarbital, ringer's solution+vitamin B+vitamin C, or placebo. We referred to these medications as conventional medicine. Because, based on our clinical experience and the collection of numerous documents, they are currently the most widely used and extensively researched medications in the clinical treatment of NVP. The specific characteristics are detailed in Table 1.

**Table 1:** Basic characteristics of the included studies.

			**Age**		**Gestation**		**Interventions**		**Dose or frequency**				
**References**	**Region**	**Sample (** ***T*** **/** ***C*** **)**	***T***	***C***	***T***	***C***	***T***	***C***	***T***	***C***	**Duration**	**Acupoint**	**Outcome**
Ensiyeh and Sakineh (24)	Iran	35/34	25.0 ± 4.2	24.2 ± 3.9	<17 (w)	<17 (w)	Ginger	Vitamin B6	1 g/d	40 mg/d	4 d	NR	Number of vomiting, total efficiency, adverse events
Pongrojpaw et al. (25)	Thailand	85/85	27.85 ± 5.3	26.38 ± 5.8	10.25 ± 2.8 (w)	9.3 ± 3.1 (w)	Ginger	Dimenhydrinate	1 g/d	100 mg/d	7 d	NR	Adverse events
Smith et al. (26)	Australia	145/146	29.6 ± 5.2	28.41 ± 5.4	8.5 ± 1.75 (w)	8.6 ± 1.75 (w)	Ginger	Vitamin B6	1,050 mg/d	75 mg/d	3 w	NR	Total efficiency
Firouzbakht et al. (27)^a^	Iran	24/35	24.9 ± 5.5	24.03 ± 3.7	9.1 ± 4.6 (w)	8.9 ± 2.9 (w)	Ginger	Vitamin B6	1,000 mg/d	160 mg/g	4 d	NR	Number of vomiting, adverse events
Firouzbakht et al. (27)^b^	Iran	24/28	24.9 ± 5.5	25.39 ± 5.24	9.1 ± 4.6 (w)	9.1 ± 3.6 (w)	Ginger	Placebo	1,000 mg/d	160 mg/g	4 d	NR	Adverse events, VAS
Mohammadbeigi et al. (28)	Iran	34/34	26.94 ± 3.94	27.88 ± 3.21	9.5 ± 2.02 (w)	10.03 ± 1.99 (w)	Ginger	Metoclopramide	600 mg/d	30 mg/d	5 d	NR	Adverse events, Rhodes
Sharifzadeh et al. (29)	Iran	28/26	28.95 ± 0.5	28.03 ± 3.7	10.9 ± 4.6 (w)	10.8 ± 4.8 (w)	Ginger	Vitamin B6	1,000 mg/d	80 mg/d	4 d	NR	Number of vomiting, Rhodes, adverse events
Chittumma et al. (30)	Thailand	61/62	23.8 ± 5.1	24.4 ± 5.3	12 ± 2 (w)	11 ± 2 (w)	Ginger	Vitamin B6	650 mg/d	15 g/d	4 d	NR	Adverse events, Rhodes
Haji Seid Javadi et al. (31)	Iran	47/48	26 ± 4	27 ± 4.2	62.9 ± 8.1 (d)	62.9 ± 8.6 (d)	Ginger	Vitamin B6	1,000 mg/d	80 mg/d	4 d	NR	Adverse events
Willetts et al. (32)	Australia	48/51	33 ± 5.25	31 ± 6.25	9 ± 3.125 (w)	9 ± 3.125 (w)	Ginger	Placebo	500 mg ginger extract /d	NR	4 d	NR	Adverse events
Ozgoli et al. (33)	Iran	32/35	24.1 ± 4.8	23.3 ± 5	13 ± 3 (w)	13 ± 3 (w)	Ginger	Placebo	1,000 mg/d	1,000 mg/d	4 d	NR	Total efficiency, adverse events
Abidah et al. (34)	Indonesia	48/48	20–35	20–35	1–12 (w)	1–12 (w)	Ginger	Placebo	2.5 g/d	10 g/d	7 d	NR	Adverse events
Zahra et al. (35)	Iran	32/30	19–35 (y)	19–35 (y)	7–17 (w)	7–17 (w)	Ginger	Placebo	2.5 g/d	2.5 g/d	4 d	NR	VAS, adverse events
Saberi et al. (36)	Iran	37/36	27.35 ± 5.93	26.85 ± 4.90	8.97 ± 0.05 (w)	9.85 ± 2.27 (w)	Ginger	Placebo	NR	NR	3 w	NR	Adverse events
Vutyavanich et al. (37)	Thailand	32/35	28.3 ± 5.8	28.6 ± 5.5	10.4 ± 2.3 (w)	10.3 ± 2.6 (w)	Ginger	Placebo	1 g/d	1 g/d	4 d	NR	VAS, adverse events
Tara et al. (38)^a^	Iran	30/30	26.0 ± 4.7	26.6 ± 5.2	9.6 ± 1.7 (w)	8.7 ± 2.2 (w)	Acupressure	Placebo	4 times/d	4 times/d	4 d	PC6, TE5	Rhodes, adverse events, total efficiency, number of vomiting
Tara et al. (38)^b^	Iran	30/30	26.0 ± 4.7	26.5 ± 4.3	9.6 ± 1.7 (w)	9.3 ± 1.3(w)	Acupressure	Vitamin B6 plus metoclopramide	4 times/d	4 times/d	4 d	PC6, TE5	Total efficiency, adverse events
Negarandeh et al. (39)	Iran	64/64	30.46 ± 5.07	29.95 ± 5.23	77.95 ± 15.61 (d)	77.34 ± 16.97 (d)	Ear acupressure	Placebo	3 times/d	3 times/d	4 d	Point zero, Sympathetic Autonomic, Shen Men, cardia	Rhodes, adverse events
Jamigorn and Phupong (40)	Thailand	33/33	28.2 ± 5.1	28.1 ± 5.6	8.1 ± 1.7 (w)	8.9 ± 3.5 (w)	Acupressure	Vitamin B6	NR	100 mg/d	7 d	PC6	Rhodes, reduction of antiemetics
Puangsricharern et al. (41)	Thailand	45/46	26.4 ± 5.6	27.0 ± 5.74	11.1 ± 2.1 (w)	11.2 ± 2.3 (w)	Ear acupressure	Oral anti-emetic drug	4 times/d	NR	4 d	On the inner surface of the auricle at the concha ridge zone	Rhodes, reduction of antiemetics
Adlan et al. (42)	Malaysia	60/60	29.0 ± 4.92	28.4 ± 4.34	9.7 ± 2.09 (w)	9.2 ± 2.03 (w)	Acupressure	Placebo	12 h/d	12 h/d	3 d	PC6	Total efficiency
Zhu et al. (43)	China	30/30	29.97 ± 2.61	30.25 ± 2.53	<12 (w)	<12 (w)	Electro acupressure	Sodium Lactate Ringer's Injection	24 h	1 time/d	4 w	PC6	Total efficiency, adverse events
Mohd Nafiah et al. (44)	Malaysia	45/45	29.3 ± 4.5	30.8 ± 4.1	10 ± 2.8 (w)	10.16 ± 2.2 (w)	Acupressure	Venous metoclopramide	3 times/d	3 times/d	1 d	NR	Total efficiency
Naeimi Rad et al. (45)	Iran	40/40	26.03 ± 4.18	25.88 ± 5.58	9.55 ± 1.81 (w)	9.45 ± 2.02 (w)	Acupressure	Placebo	2 times/d	2 times/d	4 d	NR	Adverse events
Root (46)	UK	119/112	NR	NR	11.4 ± 3.0 (w)	10.6 ± 2.3 (w)	Acupressure	Placebo	2 times/d	2 times/d	4 d	NR	Total efficiency
Heazell et al. (47)	Australia	40/40	25.4 ± 0.95	27.7 ± 0.89	8.5 ± 0.32 (w)	9.0 ± 0.36 (w)	Acupressure	Placebo	8 h/d	8 h/d	NR	NR	Adverse events
Hyde (48)	USA	8/8	NR	NR	NR	NR	Acupressure	Placebo	NR	NR	5 d	NR	Adverse events
Smith et al. (49)	Australia	148/148	30.1 ± 4.8	29.6 ± 4.6	8.3 ± 2.5 (w)	8 ± 2.25 (w)	Acupuncture	Placebo	2 times/w	2 times/w	4 w	PC6	Rhodes, total efficiency, adverse events
Carlsson et al. (50)	Sweden	17/16	28.4 ± 3.5	28.4 ± 3.5	9.9 ± 2.5 (w)	9.9 ± 2.5 (w)	Acupuncture	Placebo	3 times/d	3 times/d	8 d	PC6	Total efficiency, adverse events
Xie (51)	China	47/47	26 ± 4	27 ± 4	NR	NR	Manual acupuncture	Vitamin B	1 time/d	1 time/d	10 d	Scalp Acupuncture Stomach Area: RN12, PC6, ST36	Total efficiency
Sun (52)	China	50/50	NR	NR	NR	NR	Manual acupuncture	10% glucose+5% glucose saline+Vitamin B6+5% sodium bicarbonate+ Phenobarbital	1 time/d	3 times/day	2 w	RN12, PC6, ST36	Total efficiency
Chen and Ning (53)	China	18/18	22–34	22–34	NR	NR	Electroacupuncture	Ringer's Solution+Vitamin B+Vitamin C	1 time/d	1 time/d	5 d	PC6, CV17, CV12	Total efficiency
Knight et al. (54)	UK	28/27	30.7 ± 4.5	30.3 ± 4.5	7.8 ± 1.0 (w)	8.0 ± 1.0 (w)	Acupuncture	Placebo	2 times/w	2 times/w	2 w	ST36, SP4, CV12, PC6	Adverse events
Sinha et al. (55)	Australia	170/170	29 ± 6.67	31 ± 5.93	NR	NR	Acupressure	Placebo	24 h/d	24 h/d	NR	NR	Total efficiency
Habek et al. (56)^a^	Croatia	10/8	20.4 ± 4.7	20.8 ± 4.1	7 ± 0.75 (w)	8 ± 1.25 (w)	Manual acupuncture	Placebo	1 time/d	1 time/d	1 w	PC6	Adverse events
Habek et al. (56)^b^	Croatia	11/7	21.3 ± 3.1	22.1 ± 3.9	8 ± 1 (w)	8 ± 1.25 (w)	Acupressure	Placebo	1 time/d	1 time/d	1 w	PC6	Total efficiency, adverse events

^a, b^It represents different test and control groups for the same article.

w, week; d, day; NR, not report; T, test group; C, control group; VAS, visual analog scale.

### Risk of bias of included studies

**Figure 2:**
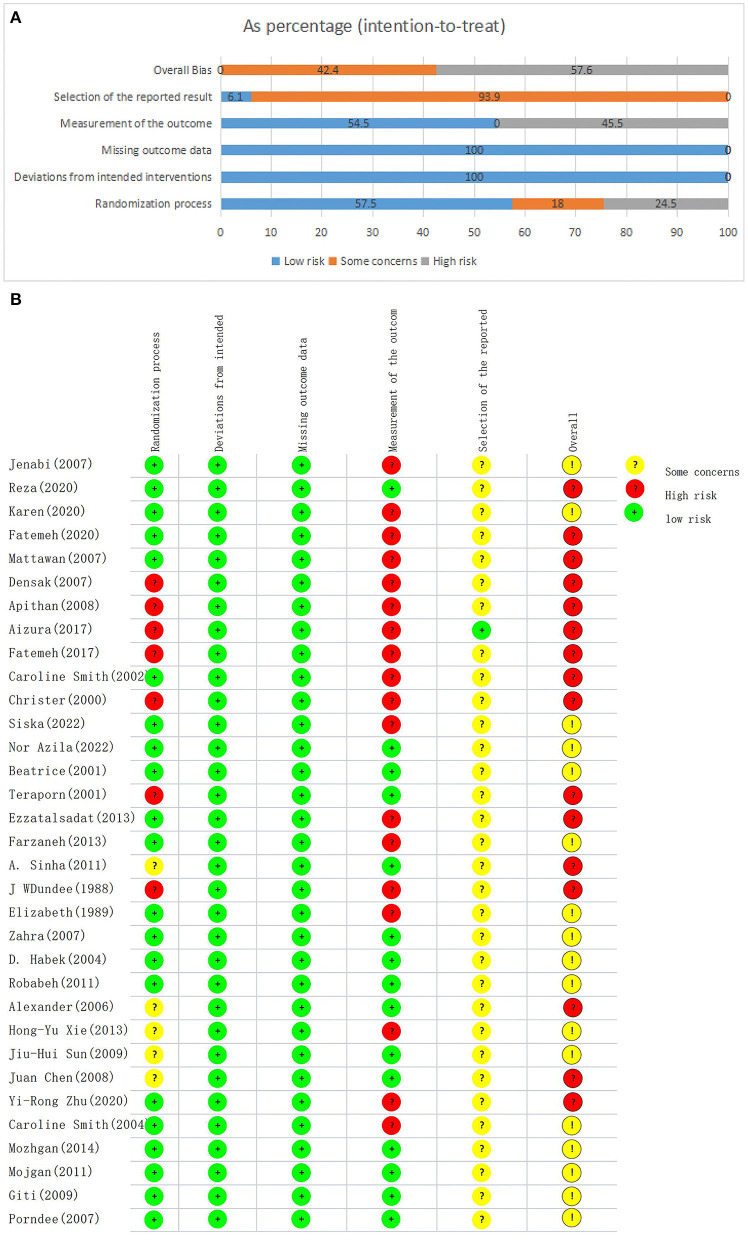
Risk of bias of RCTs **(A)** Risk of bias summary; **(B)** Risk of bias graph.

The risk of bias in the included RCTs is listed in Figure 2. Eight articles (24, 29, 31, 34, 41, 42, 48, 49) did not use allocation concealment, so they were judged as “N”, and the other five articles (43, 46, 51–53) did not mention allocation concealment, so were judged as “NI”. In terms of random sequence, the results showed that 57.5% of the articles (25–28, 30, 32, 33, 35–39, 44, 45, 47, 50, 54–56) were judged as “low risk”, 18.0% as “some concern” (40, 43, 46, 51–53) and 24.5% as “high risk” (24, 29, 31, 34, 41, 42, 48, 49). All articles did not deviate from the intended interventions and were therefore judged to be “low risk”. All articles had no data loss or data loss within acceptable limits, so they were judged to be “low risk”. For the measurement of results, the results showed that 45.5% of the articles (24–26, 29, 31, 32, 34, 36, 38, 40–43, 46, 48–51) were judged as “high risk” and 54.5% (27, 28, 30, 33, 35, 37, 39, 44, 45, 47, 52–56) as “low risk”. Thirty articles (25–28, 30–55) did not elaborate on the pre-planned or detailed full protocol, so were judged to be “NI”. For the selection of the reported result, our results showed that 93.9% of the articles (25–28, 30–56) were judged as “some concern” and 6.1% as “low risk” (24, 29). To summarize the risk assessment results of all sections, we could see that 42.4% (28, 30, 33, 35, 37, 39, 44, 45, 47, 52–56) of the articles were “some concern” and 57.6% (24–27, 29, 31, 32, 34, 36, 38, 40–43, 46, 48–51) were “high risk”.

### Effects of interventions

#### Ginger

##### Ginger vs. conventional medicine

###### Rhodes index

**Figure 3:**
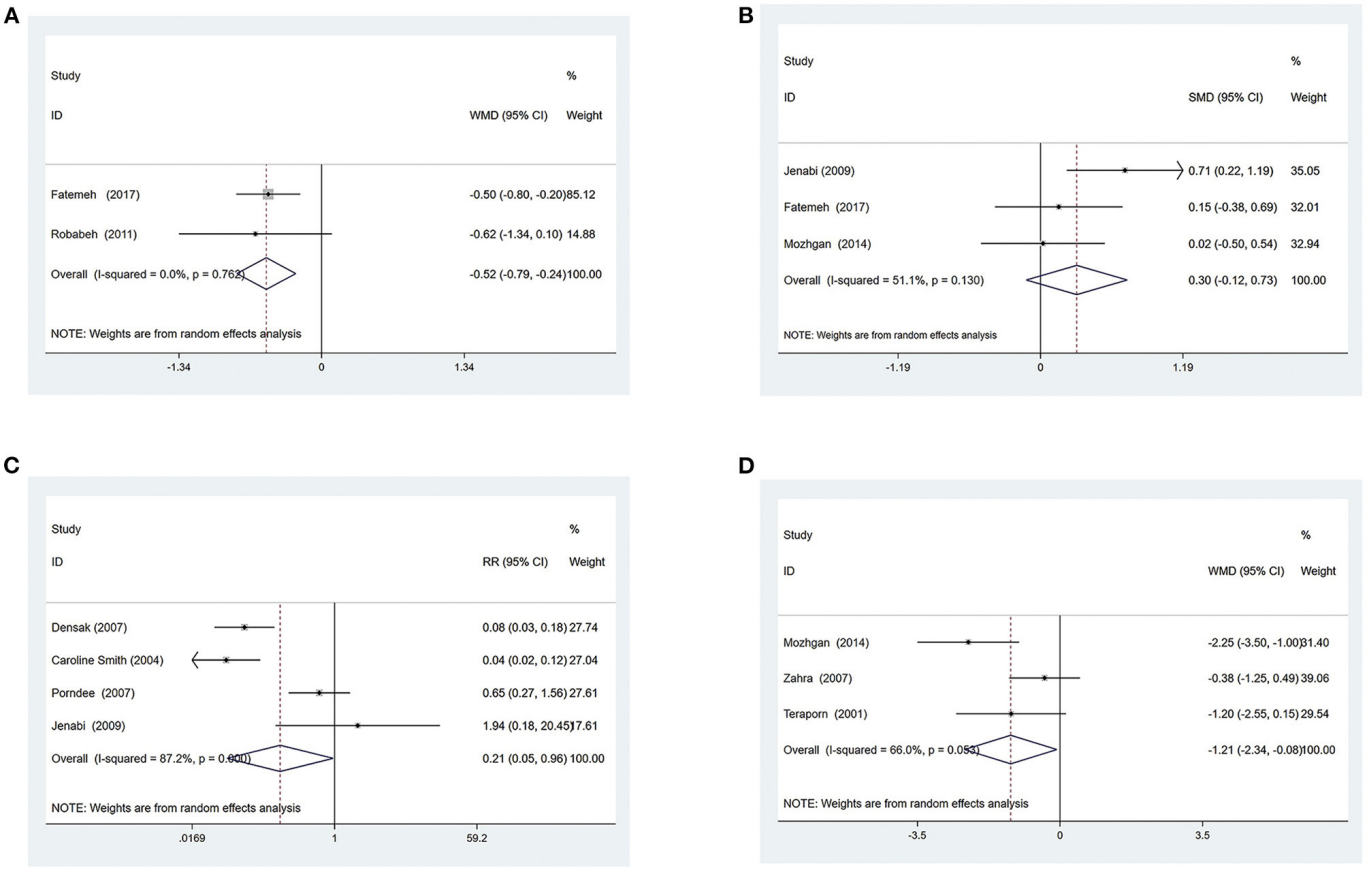
*Ginger vs. Conventional medicine* **(A)** Rhodes index; **(B)** Number of vomiting; **(C)** Adverse events *Ginger vs. Placebo group*; **(D)** VAS of Nausea.

Two trials (28, 29) compared ginger with conventional medicine, evaluating 122 patients with NVP. The pooled results showed that ginger could better relieve NVP symptoms compared to conventional medicine [WMD = −0.52, 95% CI (−0.79, −0.24), *P* ≤ 0.001] (Figure 3A), and the quality of evidence was moderate based on GRADE analysis (Supplementary material 2).

###### Number of vomiting

Three trials (24, 27, 29) compared ginger with conventional medicine, evaluating 182 patients with NVP. The pooled results showed that ginger had no obvious effect on reducing the number of vomiting for patients compared to conventional medicine [SMD = 0.30, 95% CI (−0.12, 0.73), *P* = 0.160] (Figure 3B). The GRADE analysis revealed that the overall level of quality of the evidence to support this conclusion was low (Supplementary material 2).

###### Adverse events

A total of five articles (24–27, 30) mentioned adverse events, one of which (27) reported severe NVP, stomachache, and heartburn during the treatment, but did not specify the number of people. In the remaining four studies (24–26, 30), 137 of 326 patients (42%) allocated to the ginger group experienced adverse events compared with 278 of 327 patients (85%) assigned to the control group. Side effects described in these articles included heartburn, drowsiness, belching, congenital abnormality, sedation, arrhythmia, headache, dry retching, and spontaneous abortions. By summarizing and analyzing the available data, the results showed that ginger had fewer side effects when treating NVP than conventional medicine [RR = 0.21, 95% CI (0.05, 0.96), *P* = 0.045] (Figure 3C). The quality of evidence was low based on GRADE analysis (Supplementary material 2).

##### Ginger vs. the placebo group

###### VAS of nausea

Three trials (27, 35, 37) compared ginger with a placebo, evaluating 181 patients with NVP. The pooled results showed that ginger had more significant effects on patients' nausea symptoms compared to the placebo group [WMD = −1.21, 95% CI (−2.34, −0.08), *P* = 0.036] (Figure 3D). The GRADE analysis revealed that the overall level of quality of the evidence to support this conclusion was low (Supplementary material 2).

###### Number of vomiting

**Figure 4:**
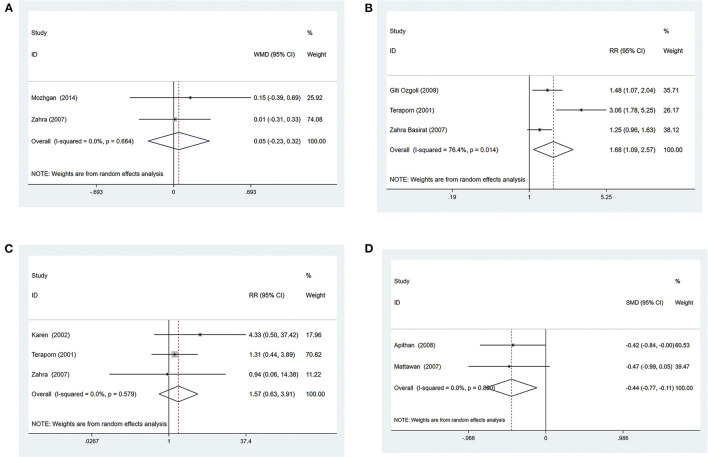
*Ginger vs. Placebo group* **(A)** Number of vomiting; **(B)** Effective rate; **(C)** Adverse events *Acupressure vs. Conventional medicine* **(D)** Dosage of antiemetic drugs.

Two trials (27, 35) compared ginger with a placebo, evaluating 114 patients with NVP. The pooled results showed that ginger had no obvious effect on reducing the number of vomiting of patients compared to the placebo [WMD = 0.05, 95% CI (−0.23, 0.32), *P* = 0.743] (Figure 4A). The quality of evidence was low based on GRADE analysis (Supplementary material 2).

###### Effective rate

Three trials (33, 35, 37) compared ginger with a placebo, evaluating 196 patients with NVP. The pooled results showed that the effective rate was higher compared with the placebo when patients received ginger [RR = 1.68, 95% CI (1.09, 2.57), *P* = 0.018] (Figure 4B), and the quality of evidence was low based on GRADE analysis (Supplementary material 2).

###### Adverse events

A total of 6 articles (27, 32, 33, 35–37) mentioned adverse events, evaluating 420 patients with NVP, one of which (27) reported stomachache and heartburn during the treatment, but did not specify the number of affected individuals. Another article (36) mentioned heartburn, but there was also no specific number of cases. One article (33) clearly stated that participants had no adverse effects. In the remaining three studies (32, 35, 37), 18 of 112 patients (16%) allocated to the ginger group experienced adverse events compared with 10 of 116 patients (9%) assigned to the control group. The available data were summarized and analyzed and the results showed that side effects of ginger were equivalent to those of placebo when treating NVP [RR = 1.57, 95% CI (0.63, 3.91), *P* = 0.336] (Figure 4C). The GRADE analysis revealed that the overall level of quality of the evidence to support this conclusion was very low (Supplementary material 2).

#### Acupressure

##### Acupressure vs. conventional medicine

###### Dosage of antiemetic drugs

Two trials (40, 41) compared acupressure with conventional medicine, evaluating 151 patients with NVP. The pooled results showed that acupressure could further reduce the number of antiemetic drugs used by patients compared with conventional medicine [SMD = −0.44, 95% CI (−0.77, −0.11), *P* = 0.008] (Figure 4D). The GRADE analysis revealed that the overall level of quality of the evidence to support this conclusion was low (Supplementary material 2).

###### Effective rate

**Figure 5:**
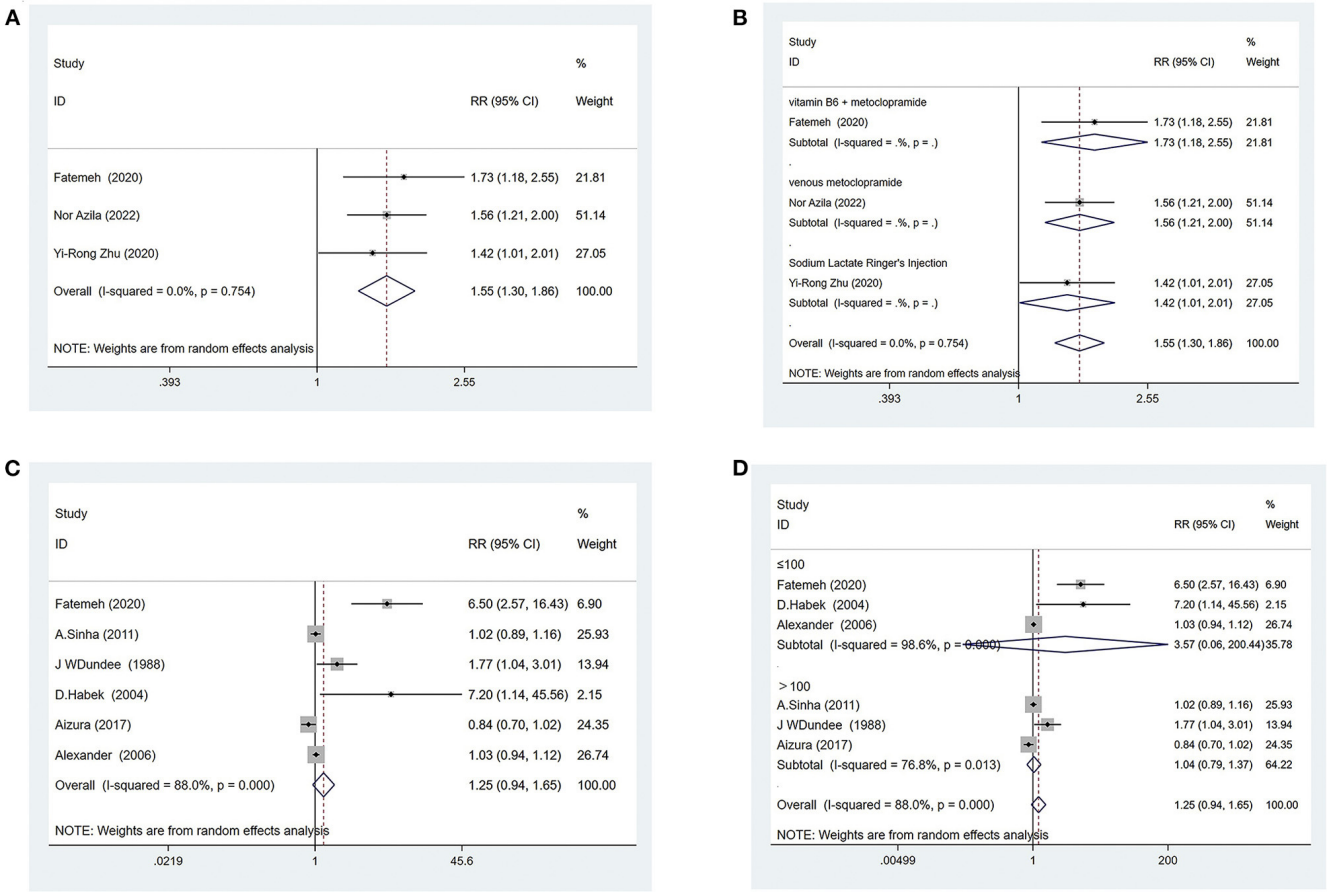
*Acupressure vs. Conventional medicine* **(A)** Effective rate; **(B)** Subgroup analysis based on different medications; *Acupressure vs. Placebo group*; **(C)** Effective rate; **(D)** subgroup analysis based on sample size.

Three trials (38, 43, 44) compared acupressure with conventional medicine, evaluating 210 patients with NVP. The pooled results showed that the effective rate was higher when patients received acupressure compared with conventional medicine [RR = 1.55, 95% CI (1.30, 1.86), *P* ≤ 0.001] (Figure 5A). The GRADE analysis revealed that the overall level of quality of the evidence to support this conclusion was low (Supplementary material 2).

For further analysis, we grouped the different types of medications for subgroup analysis. The results showed that acupressure was superior to vitamin B6 + metoclopramide [RR = 1.73, 95% CI (1.18, 2.55), *P* = 0.005] (Figure 5B), venous metoclopramide [RR = 1.56, 95% CI (1.21, 2.00), *P* = 0.001] (Figure 5B) and sodium lactate ringer's injection [RR = 1.42, 95% CI (1.01, 2.01), *P* = 0.046] (Figure 5B).

###### Adverse events

A total of five articles (38, 40, 41, 43, 44) evaluating 367 patients with NVP mentioned no adverse events.

##### Acupressure vs. the placebo group

###### Effective rate

Six trials (38, 42, 46, 47, 55, 56) compared acupressure with placebo, evaluating 849 patients with NVP. The pooled results showed that acupressure had the same effect as placebo at the effective rate [RR = 1.25, 95% CI (0.94, 1.65), *P* = 0.124] (Figure 5C), and the quality of evidence was low based on GRADE analysis (Supplementary material 2).

To find sources of heterogeneity, subgroup analyses were conducted for sample size, as well as year separately, and it was found that the results did not change whether the sample size was >100 or <100 (Figure 5D). The results also did not change depending on whether the year of publication was before or after 2010 (Supplementary material 3).

###### Adverse events

A total of 9 articles (38, 39, 42, 45–48, 55, 56) mentioned adverse events, evaluating 1,073 patients with NVP, eight of which (38, 39, 42, 45, 46, 48, 55, 56) did not mention adverse effects. One paper (47) reported side effects in which one miscarriage occurred in a trial group of 40 patients, yet two cases occurred in a control group with the same number of patients. These could not be combined for analysis because too little data were available. However, this article showed that the safety of acupressure was better than traditional medicine.

#### Acupuncture

##### Acupuncture vs. conventional medicine

###### Effective rate

**Figure 6:**
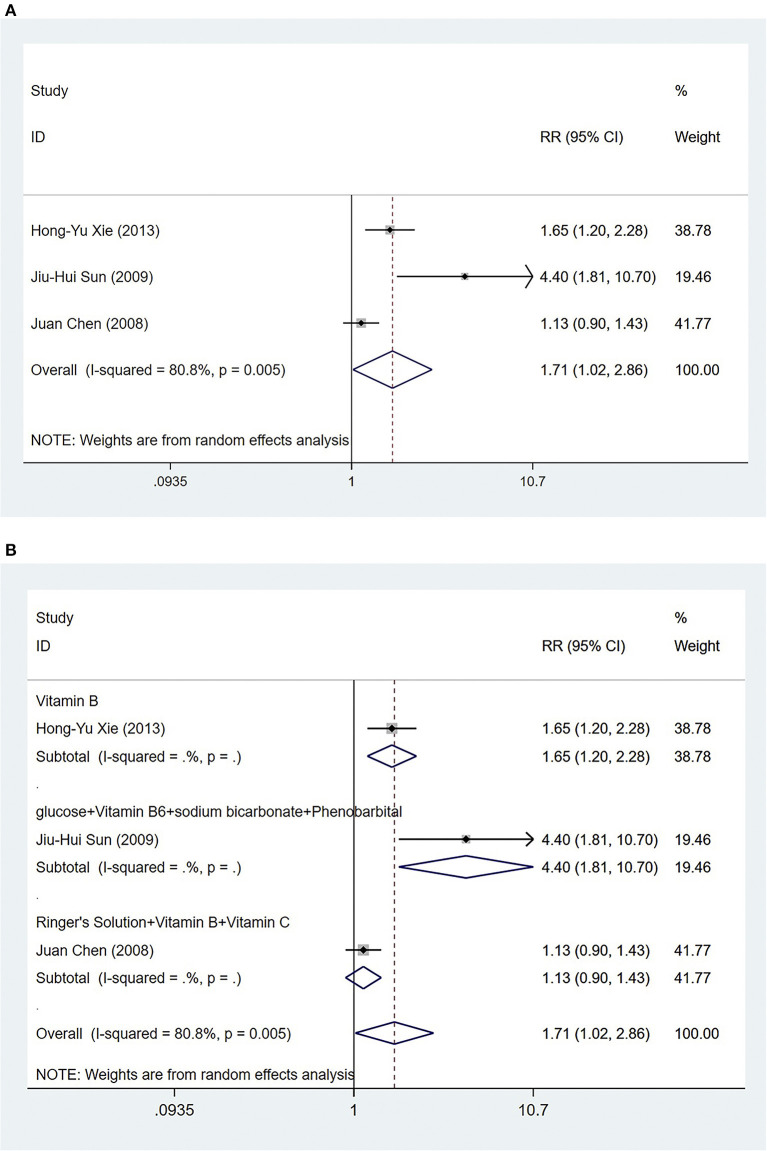
*Acupuncture vs. Conventional medicine:* **(A)** Effective rate; **(B)** Subgroup analysis based on different medications.

Three trials (51–53) compared acupuncture with conventional medicine, evaluating 230 patients with NVP. The pooled results showed that the effective rate was higher compared with conventional medicine when patients received acupuncture [RR = 1.71, 95% CI (1.02, 2.86), *P* = 0.042] (Figure 6A). The GRADE analysis revealed that the overall level of quality of the evidence to support this conclusion was low (Supplementary material 2).

For further analysis, we grouped the different types of medications for subgroup analysis. The results showed that acupuncture treatment was more effective than vitamin B [RR = 1.65, 95% CI (1.20, 2.28), *P* = 0.002] (Figure 6B). Compared with glucose+vitamin B6+sodium bicarbonate+phenobarbital, acupuncture treatment had a higher effective rate [RR = 4.40, 95% CI (1.81, 10.70), *P* = 0.001] (Figure 6B). Acupuncture treatment was as effective as ringer's solution+vitamin B+vitamin C [RR = 1.13, 95% CI (0.90, 1.43), *P* = 0.297] (Figure 6B).

###### Adverse events

A total of three articles (51–53) evaluating 230 patients with NVP mentioned no adverse events.

##### Acupuncture vs. placebo group

###### Adverse events

A total of four articles (49, 50, 54, 56) mentioned adverse events, evaluating 402 patients with NVP, two of which (49, 56) did not mention adverse effects. One article (50) clearly stated that participants had no adverse effects. One paper (54) reported 11 adverse events in the trial group, including fatigue, sleep disturbance, and arm heaviness, and 8 adverse events in the same number of controls, including vomiting, flatulence, dreaming, and a feeling of coldness, which seemed to indicate that acupuncture had similar safety profile to placebo.

### Sensitivity analysis and publication bias

**Figure 7:**
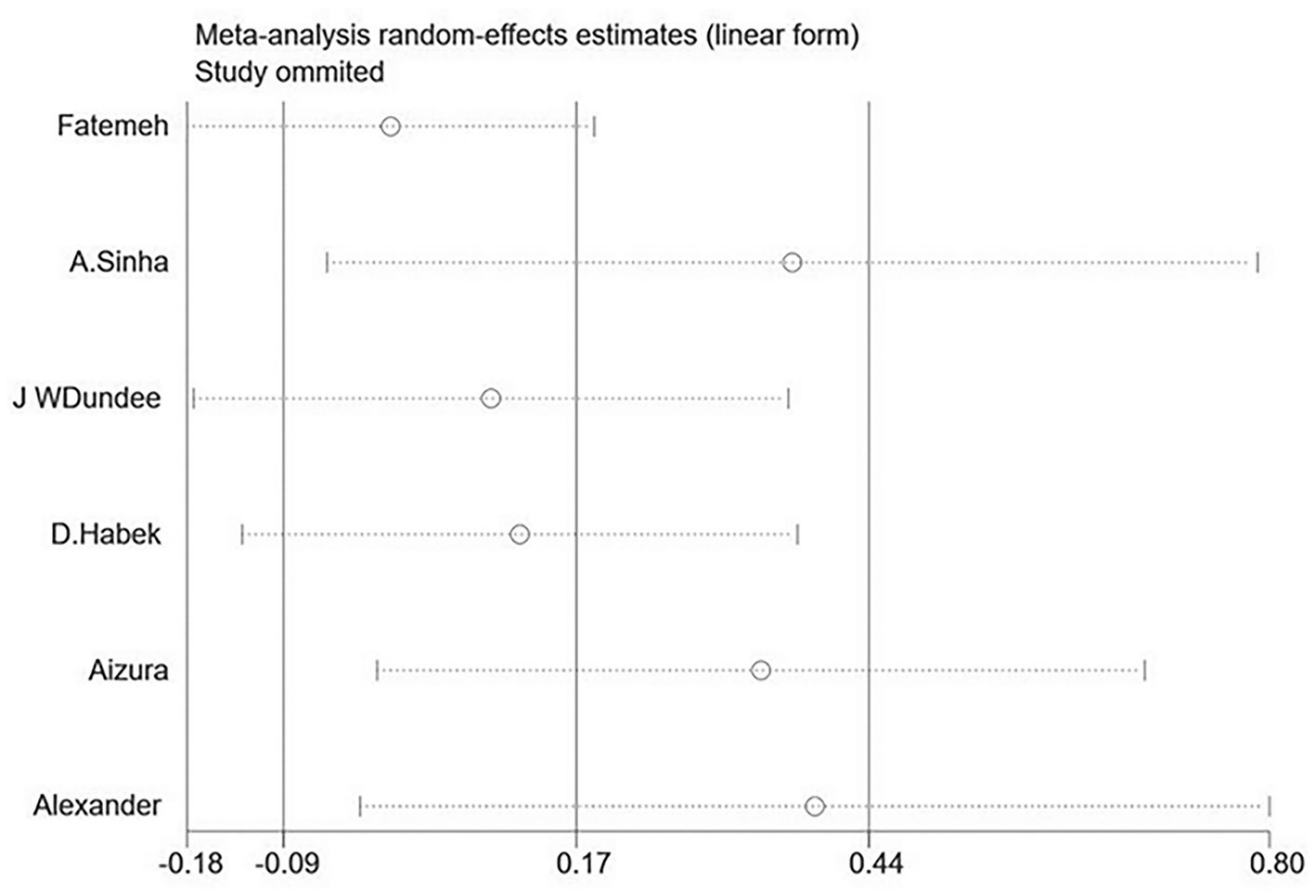
*Sensitivity analysis:* Effective rate of Acupressure vs. Placebo group.

Sensitivity analysis (Figure 7) showed that no single study significantly affected the results, reflecting that the results were statistically robust.

A funnel analysis and Begg's test were not performed due to limitations of the number of articles in the *per* outcome.

### Level of overall evidence

Table 2 presents the quality evaluation of each outcome of CAM on NVP.

**Table 2:** The outcome of the meta-analysis.

**Outcomes**	**Number**	**T/C**	**End of treatment**					
			**WMD/RR/SMD**	**95%CI**	**Heterogeneity**	***P*** **-value**	**F/R**	**GRADE**
**Ginger vs. placebo**								
VAS	3	88/100	WMD = −1.21	(−2.34)–(−0.08)	66.0%	*P* = 0.036	R	Low
Adverse events	3	112/116	RR = 1.57	(0.63)–(3.91)	0.0%	*P* = 0.336	R	Very low
Number of vomiting	2	56/65	WMD = 0.05	(−0.23)–(0.32)	0.0%	*P* = 0.743	R	Low
Effective rate	3	96/100	RR = 1.68	(1.09)–(2.57)	76.4%	*P* = 0.018	R	Low
**Ginger vs. CM**								
Number of vomiting	3	87/95	SMD = 0.30	(−0.12)–(0.73)	51.1%	*P* = 0.160	R	Low
Rhodes index	2	62/60	WMD = −0.52	(−0.79)–(−0.24)	0.0%	*P* ≤ 0.001	R	Moderate
Adverse events	4	76/77	RR = 0.21	(0.05)–(0.96)	87.2%	*P* = 0.045	R	Low
**AP vs. placebo**								
Effective rate	6	429/420	RR = 1.25	(0.94)–(1.65)	88.0%	*P* = 0.124	R	Low
**AP vs. CM**								
Effective rate	3	105/105	RR = 1.55	(1.30)–(1.86)	0.0%	*P* ≤ 0.001	R	Low
Number of anti-emetic drug	2	75/76	SMD = −0.44	(−0.77)–(−0.11)	0.0%	*P* = 0.008	R	Low
**AT vs. CM**								
Effective rate	3	115/115	RR = 1.71	(1.02)–(2.86)	80.8%	*P* = 0.042	R	Low

WMD, weighted mean difference; SMD, standard mean difference; RR, risk ratio; CI, confidence interval; VAS, visual analog scale; P, P-value represents clinical significance; CM, conventional medicine; AP, acupressure treatment; AT, acupuncture treatment; T, test group; C, control group; F, Fixed effects model; R, Random effects model; GRADE, Grades of Recommendation, Assessment, Development, and Evaluation; Very low, very low quality; Low, low quality; Moderate, Moderate quality.

High quality: Further research is very unlikely to change our confidence in the estimate of effect. Moderate quality: Further research is likely to have an important impact on our confidence in the estimate of effect and may change the estimate. Low quality: Further research is very likely to have an important impact on our confidence in the estimate of effect and is likely to change the estimate. Very low quality: We are very uncertain about the estimate.

In the comparison of ginger with conventional medicine, the quality of evidence suggested that the Rhodes in the outcome indicators was of moderate quality, while the number of vomiting and adverse events was of low quality. The evidence for the comparison of ginger with placebo was variable in VAS (low), number of vomiting (low), effective rate (low), and adverse events (very low).

In the comparison of acupuncture with conventional medicine, the quality of evidence suggested that the effective rate in the outcome indicators was of low quality. Evidence for the comparison of acupressure with conventional medicine was variable in the dosage of antiemetic drugs (low) and effective rate (low).

In the comparison of acupressure with placebo, the quality of evidence suggested that the effective rate in the outcome indicators was of low quality.

## Discussion

### Summary of results

The results showed that ginger could improve patients' Rhodes index compared with conventional medicine, with fewer side effects but no significant improvement in the number of vomiting. Ginger improved nausea symptoms more than placebo and was more effective, but the improvement in vomiting was not obvious. Compared with conventional medicine, acupressure could reduce the amount of vomiting medication a person takes and be more effective. Compared with a placebo, acupressure was more effective and had the same side effects. Acupuncture was more effective in treating NVP than conventional medication and placebo. Acupuncture had a similar safety profile to a placebo. The specific results of the meta-analysis are shown in Tables 2, 3.

**Table 3:** Subgroup analysis results.

**Outcome**	**Subgroup**	**Studies**	**Patients**	***RR***	**95% CI**	**Heterogeneity**	***P*-value**	**F/R**
**Effective rate**	**Types of medications (AP vs.CM)**							
	Vitamin B6 + metoclopramide	1	60	*RR* = 1.73	(1.18–2.55)	NR	*P* = 0.005	R
	Venous metoclopramide	1	90	*RR* = 1.56	(1.21–2.00)	NR	*P* = 0.001	R
	Sodium lactate ringer's injection	1	60	*RR* = 1.42	(1.01–2.01)	NR	*P* = 0.046	R
**Effective rate**	**Sample size (AP vs. placebo)**							
	≤ 100	3	158	*RR* = 3.57	(0.06–200.44)	98.60%	*P* = 0.536	R
	>100	3	691	*RR* = 1.04	(0.79–1.37)	76.80%	*P* = 0.781	R
**Effective rate**	**Publication year (AP vs. placebo)**							
	>2010	3	520	*RR* = 1.28	(0.79–2.06)	91.00%	*P* = 0.313	R
	<2010	3	329	*RR* = 1.93	(0.50–7.40)	94.40%	*P* = 0.340	R
**Effective rate**	**Types of medications (AT vs.CM)**							
	Vitamin B	1	94	*RR* = 1.65	(1.20–2.28)	NR	*P* = 0.002	R
	Glucose+Vitamin B6+sodium bicarbonate+Phenobarbital	1	100	*RR* = 4.40	(1.81–10.70)	NR	*P* = 0.001	R
	Ringer's solution+vitamin B+vitamin C	1	36	*RR* = 1.13	(0.90–1.43)	NR	*P* = 0.297	R

RR, risk ratio; F, Fixed effects model; R, Random effects model; CI, confidence interval; NR, not report; AP, acupressure; AT, acupuncture treatment; CM, conventional medicine.

### Discussion of CAM vs. conventional medicine

The results showed that ginger is as effective as conventional medicine in treating NVP, which remains consistent with the meta-results of Estelle et al. (16). Ginger can protect the gastric mucosa, promote gastrointestinal motility and block gastrointestinal adverse reactions, which has a good effect on nausea and vomiting (57). Fatemeh et al. studied pregnant women in the 6–16 weeks' gestation period and found that ginger and vitamin B6 were equivalent in improving NVP (29). Mattawan randomly divided pregnant women into a test and a control group with 85 people each (40). It was concluded that ginger was as effective as diphenhydramine in treating nausea and vomiting during pregnancy (40). The above studies were consistent with the results of this meta-analysis.

The conventional medicines mainly included in this article included diphenhydramine, vitamin B6, metoclopramide, and so on. Among them, diphenhydramine has the effect of antihistamine H1 receptor and has a strong inhibitory effect on the central nervous system (58). Metoclopramide has a solid central antiemetic and gastrointestinal excitatory effect (59). Combined with the above results, ginger positively impacts NVP compared with conventional medicine. Considering that ginger has fewer side effects, it is an excellent complementary replacement therapy when patients are not well treated with traditional drugs or are unwilling to take it because of concerns about side effects.

Combined with the full-text results, acupuncture, and acupressure were more effective in treating NVP than conventional medicine and there was a high level of safety. Acupuncture and pressure points are similar, mostly PC6, TE5, N12, ST36, CV17, CV12, and SP4. Among them, PC6 was the most commonly used infusion point and Chinese medicine believes that stimulating and pressing this point has a good effect on relieving NVP. Vickers reviewed 16 RCTs and found that the stimulation of PC6 was an effective antiemetic based on consistent results found by researchers using various acupuncture techniques in different patient populations (60). O'Brien et al. subjected 161 symptomatic volunteers to bilateral PC6 acupoint stimulation, bilateral sham acupoint stimulation, or a no-banding control condition. Nausea and vomiting were assessed by self-report every 12 h. Of the 161 women, 149 (92.5%) completed the protocol and reported a significant decrease in NVP (61), which was consistent with the results of this meta-analysis. This may be related to the fact that PC6 concentrates its action on the AP *via* the vagal pathway to potentially affect the areal postrema by reducing hormone release (62). One study found that electroacupuncture of PC6 by the intravenous drip of pressing significantly reduced the number of NVPs and inhibited retrograde peristaltic contractions (63). From the above results, it can be seen that acupuncture or acupressure is a good choice when the patient does not want to take any medication, even ginger.

To further study specific drugs, we performed subgroup analyses of the medications. The results showed that acupressure was superior to vitamin B6 + metoclopramide, venous metoclopramide, and sodium lactate ringer's injection. Acupuncture treatment was more effective than vitamin B and glucose+vitamin B6+sodium bicarbonate+Phenobarbital. Acupuncture treatment was as effective as ringer's solution+vitamin B+vitamin C. It is worth noting that due to the limitation of the number of relevant literature, the results need to be treated with caution. More clinical trials will be needed in the future to verify this result.

### Discussion of CAM vs. placebo

Placebo measures included stimulating non-acupuncture points, taking nitroglycerin, or using flour. In general, CAM was superior to placebo for NVP. Combined with the comparison of CAM and conventional medicine, it can be concluded that the effect of CAM on NVP is definite. As for the security of CAM, the results showed that it was close to a placebo, which also proves the high level of security of CAM.

For the high heterogeneity of acupressure vs. placebo for NVP, a subgroup analysis of sample size and year of publication was performed, which showed the same result regardless of whether the year of publication was before or after 2010 and whether the sample size was greater or <100. By reading these articles, it was discovered that some articles stimulated only the PC6. In contrast, some stimulated other points in addition to the PC6, and not all with the same frequency and treatment periods, which may also contribute to the heterogeneity. Due to the limitation of the number of articles, the frequency, acupuncture points, and treatment duration in further subgroups were not analyzed. Further large-sample RCTs are needed to verify this in the future.

### Strength and limitation

Firstly, this is the first meta-analysis to examine CAM for NVP, ensuring accuracy while comprehensively including all RCTs. Secondly, a comprehensive set of outcome indicators to evaluate the efficacy of CAM for NVP in comprehensive aspects were included. Finally, a placebo control was included, and more accurately and objectively analyzed the therapeutic effects of CAM. However, this study has the following limitations: (1) the RCT included in this paper may be sources of heterogeneity due to the different frequency and types of acupuncture, the different experiences of acupuncturists, the different amounts of acupoints and ginger pressed; (2) it must be considered that the degree of vomiting varies by trimester. However, further exploration of the efficacy of CAM for treating different degrees of vomiting in different trimesters was not conducted due to the limitation of the number of included articles; (3) through a comprehensive search, it was found that the number of articles reporting CAM for NVP is still low, the sample size is small and since there were some results with a relatively large confidence interval, false positive results may occur; and (4) although we searched eight databases comprehensively, the languages were limited to Chinese and English and the studies included were mostly conducted in Asia, which may have led to some limitations regarding population outreach.

## Conclusion

The current evidence showed that CAM was superior to placebo or conventional medicine with fewer side effects. Therefore, it is recommended that clinicians use CAM for NVP. However, due to the small number of RCTs and the small sample size, more rigorously designed, large-sample multicenter RCTs will be needed to prove these results in the future.

## Author contributions

Conception and design of the study: M-YT, S-HS, and QZ. Data collection and analysis: M-YT, R-LL, and QZ. Draft paper: M-YT, S-HS, R-LL, and QZ. Approve the final paper to be published: All authors.
