# Supplementary material for: The efficacy and safety of complementary and alternative medicine in the treatment of nausea and vomiting during pregnancy: A systematic review and meta-analysis
Source: Front Public Health. 2023 Mar 9;11:1108756. doi: 10.3389/fpubh.2023.1108756 (PMC10035790; doi:10.3389/fpubh.2023.1108756)
Supplement: Supplementary file 1 [file Table_1.DOC]

**Supplementary materials 1 Search strategy**

**Pubmed**

1. "Acupuncture"[Mesh]
2. ((acupuncture[Title/Abstract]) OR (electroacupuncture[Title/Abstract])) OR (Pharmacopuncture[Title/Abstract])
3. #1 or #2
4. "Acupressure"[Mesh]
5. (((acupressure[Title/Abstract]) OR (Shiatsu[Title/Abstract]) OR (Chih Ya[Title/Abstract]) OR (Zhi Ya[Title/Abstract])))
6. #4 or #5
7. "ginger"[Mesh]
8. (ginger[Title/Abstract]) OR (Zingiber officinale[Title/Abstract])
9. #7 or #8
10. "Complementary Therapies"[Mesh]
11. ((Complementary Therapies[Title/Abstract]) OR (Alternative Medicine[Title/Abstract])) OR (Alternative Therapies[Title/Abstract])
12. #10 or #11
13. #3 or #6 or #9 or #12
14. "Vomiting"[Mesh]
15. ((Vomiting[Title/Abstract]) OR (Emesis[Title/Abstract]))
16. #14 or #15
17. "Nausea"[Mesh]
18. (Nausea [Title/Abstract])
19. #17 or #18
20. #16 or #19
21. "Pregnant Woman"[Mesh]
22. Pregnant Woman [Title/Abstract]
23. "Pregnancy"[Mesh]
24. ((Pregnancy[Title/Abstract]) OR (Pregnancies[Title/Abstract])) OR (Gestation[Title/Abstract]))
25. #21 or #22 # or #23 or #24
26. #13 and #20 and #25

**Web of science**

1.Acupuncture (主题) or Pharmacopuncture (主题) or Electroacupuncture (主题)

2.Acupressure (主题) or Shiatsu (主题) or Chih Ya (主题)

3.ginger (主题) or Zingiber officinale (主题)

4.complementary therapies (主题) or alternative medicine (主题) or (alternative therapies (主题)

5.vomiting (主题) or emesis (主题)

6.Nausea (主题)

7.#1 or #2 or #3 or #4

8.#5 or #6

9.Pregnant Women (主题) or Pregnancy (主题) or Pregnancies (主题) or Gestation (主题)

10.#7 and #8 and #9

**Cochrane**

1. MeSH descriptor: [Acupuncture] explode all trees

2. (Acupuncture):ti,ab,kw OR (Pharmacopuncture):ti,ab,kw OR (Electroacupuncture):ti,ab,kw

3. #1 or #2

4. MeSH descriptor: [Acupressure] explode all trees

5. (acupressure):ti,ab,kw OR (Shiatsu):ti,ab,kw OR (Chih Ya):ti,ab,kw

6. #4 OR #5

7. MeSH descriptor: [Ginger] explode all trees

8. (ginger):ti,ab,kw OR (Zingiber officinale):ti,ab,kw

9. #7 OR #8

10. #3 OR #6 OR #9

11. MeSH descriptor: [Vomiting] explode all trees

12. (Emesis):ti,ab,kw

13. #11 or #12

14. MeSH descriptor: [Nausea] explode all trees

15. #13 OR #14

16. MeSH descriptor: [Complementary Therapies] explode all trees

17. (Complementary Therapies):ti,ab,kw OR (Alternative Medicine):ti,ab,kw OR (Alternative Therapies):ti,ab,kw

18. #16 OR #17

19. MeSH descriptor: [Pregnant Women] explode all trees

20. (Pregnant Women):ti,ab,kw

21. MeSH descriptor: [Pregnancy] explode all trees

22. ("pregnancy"):ti,ab,kw OR (Pregnancies):ti,ab,kw AND (Gestation):ti,ab,kw

23. #19 OR #20 OR #21 OR #22

24. #10 OR #18

25. #15 AND #23 AND #24

**EMBASE**

1 'acupuncture'/exp

2 acupuncture:ab,ti OR pharmacopuncture:ab,ti OR electroacupuncture:ab,ti

3 #1 OR #2

4 'acupressure'/exp

5 acupressure:ab,ti OR Shiatsu:ab,ti OR Chih Ya:ab,ti

6 #4 OR #5

7 'ginger'/exp

8 ginger:ab,ti OR Zingiber officinale:ab,ti

9 #7 OR #8

10 'complementary therapies'/exp

11 complementary therapies:ab,ti OR alternative Medicine:ab,ti OR alternative therapies:ab,ti

12 #10 OR #11

13 #3 OR #6 OR #9 OR #12

14 'vomiting'/exp

15 vomiting:ab,ti OR emesis:ab,ti OR emesia:ab,ti OR vomitus:ab,ti

16 #14 OR #15

17 nausea:ab,ti

18 #16 OR #17

19 'pregnant woman'/exp

20 'pregnancy'/exp

21 pregnancy:ab,ti OR 'child bearing':ab,ti OR gestation:ab,ti OR gravidity:ab,ti OR 'labor 12 presentation':ab,ti

22 #19 OR #20 OR #21

23 #13 AND #18 AND #22

**VIP**

1.FT =(‘针灸’+’针’+’耳针’+’指压’)AND FT =(‘恶心呕吐’) AND FT =(‘孕妇’)

2.AB=(‘针灸’+’针’+’耳针’+’指压’) AND AB =(‘恶心呕吐’) AND AB =(‘孕妇’)

3.TI=(‘针灸’+’针’+’耳针’+’指压’)AND TI =(‘恶心呕吐’) AND TI =(‘孕妇’)

**Wanfang**

1.FT =(‘针灸’+’针’+’耳针’+’指压’) AND FT =(‘恶心呕吐’) AND FT =(‘孕妇’)

2.AB=(‘针灸’+’针’+’耳针’+’指压’) AND AB =(‘恶心呕吐’) AND AB =(‘孕妇’)

3.TI=(‘针灸’+’针’+’耳针’+’指压’)AND TI =(‘恶心呕吐’) AND TI =(‘孕妇’)

**CNKI**

1. FT =(‘针灸’+’针’+’耳针’+’指压’) AND FT =(‘恶心呕吐’) AND FT =(‘孕妇’)
2. AB=(‘针灸’+’针’+’耳针’+’指压’) AND AB =(‘恶心呕吐’) AND AB =(‘孕妇’)
3. TI=(‘针灸’+’针’+’耳针’+’指压’)AND TI =(‘恶心呕吐’) AND TI =(‘孕妇’)

**SinoMed**

("恶心呕吐"[常用字段:智能]) AND("孕妇"[常用字段:智能]) AND ("针灸"[全部字段:智能] OR "耳针"[全部字段:智能] OR "针"[全部字段:智能] OR "指压"[全部字段:智能])

Supplementary materials 2: The overall evidence quality for outcome measure

Ginger vs. placebo


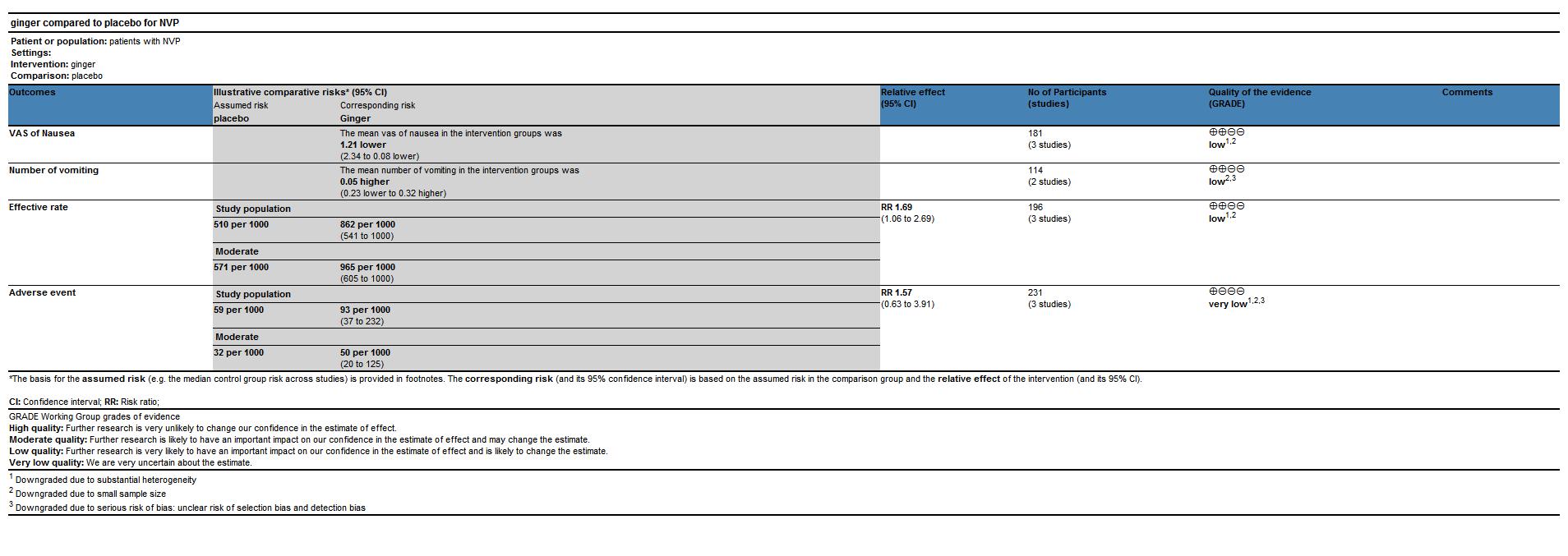


Ginger vs. CM


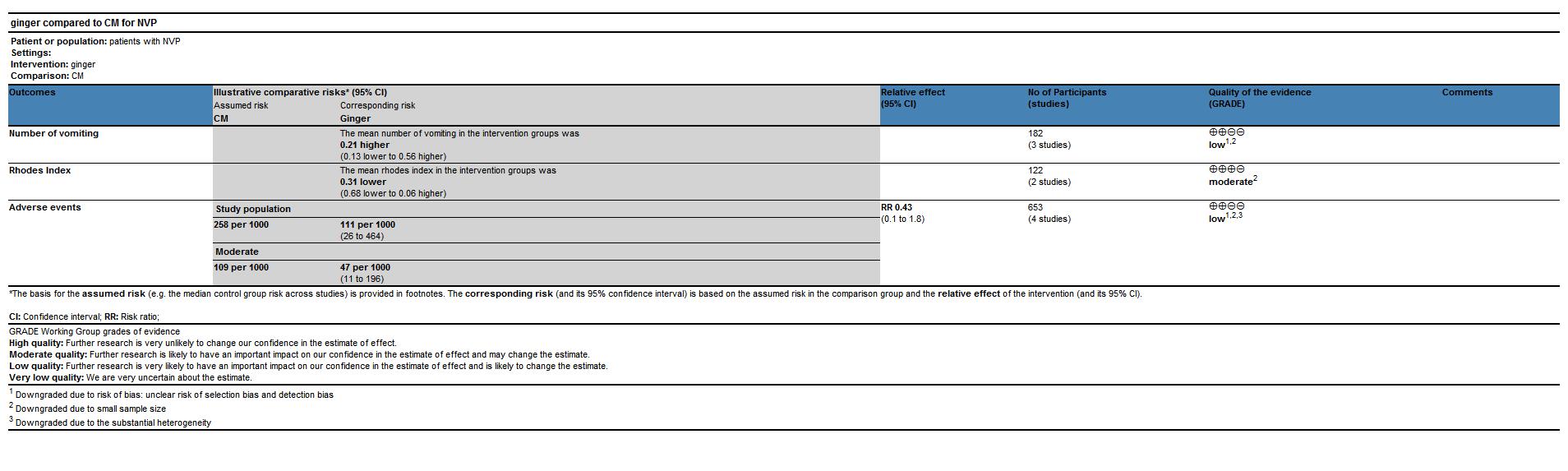


AP vs. CM


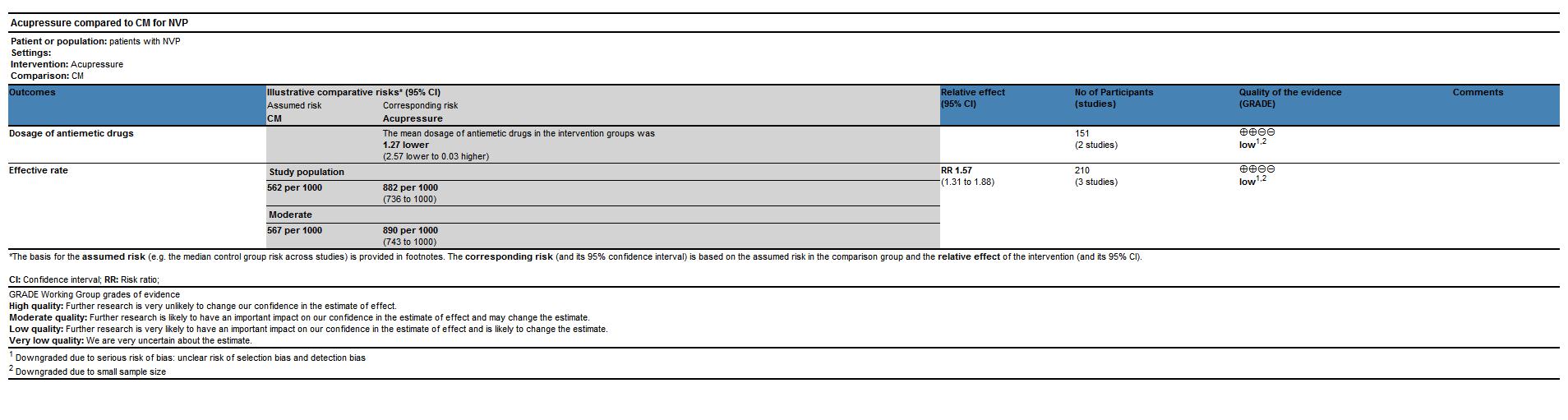


AP vs. placebo


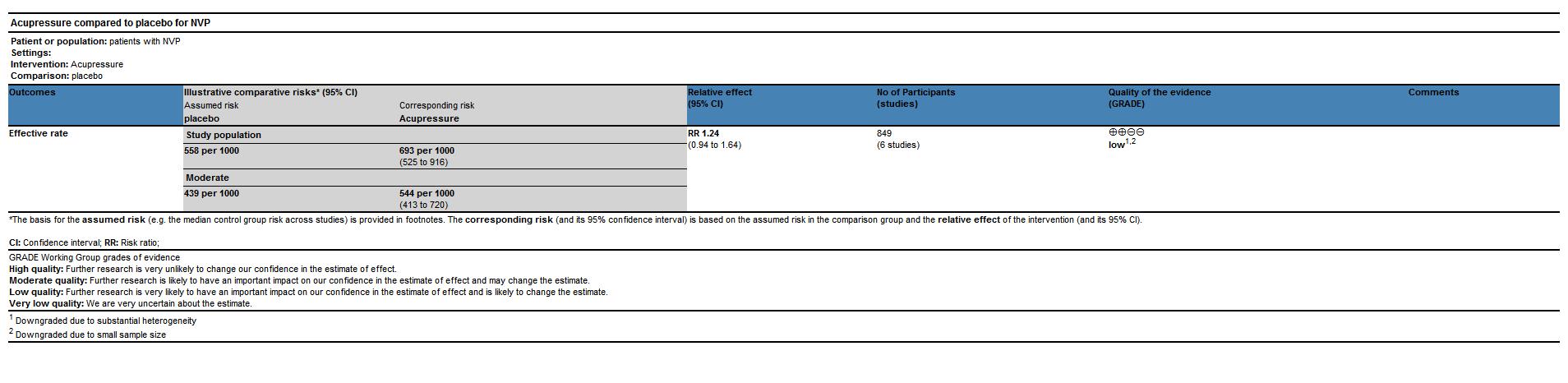


AT vs. CM


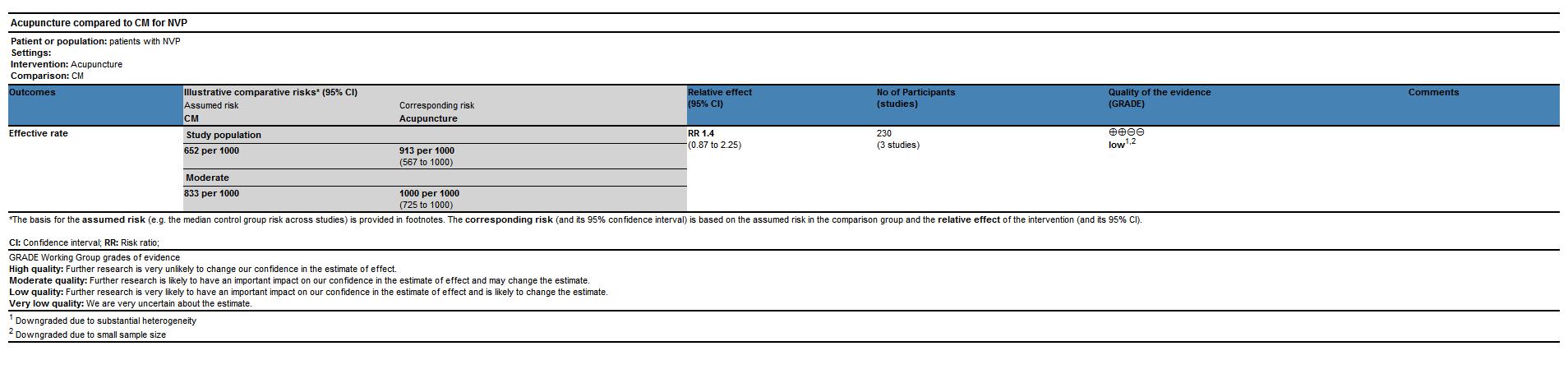


Abbreviation: **AT** :acupuncture treatment; **AP**: acupressure; **CM**: conventional medicine

Supplementary materials 3: *Acupressure versus. Placebo group:* Subgroup analysis based on publication year


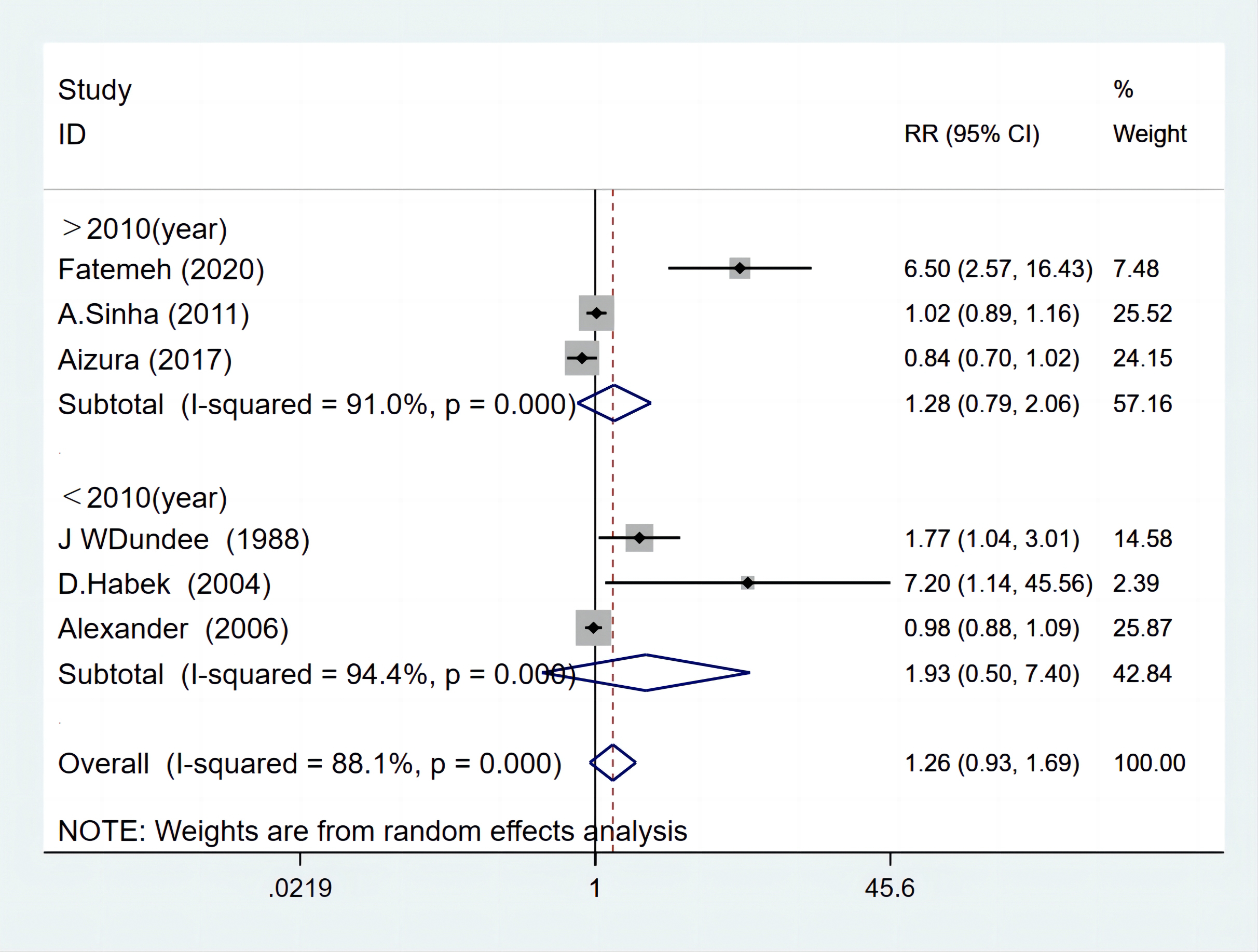


*Acupressure versus. Placebo group:* Subgroup analysis based on publication year

Supplementary materials 4: PRISMA checklist

| **Section and Topic** | **Item #** | **Checklist item** | **Location where item is reported** |
| --- | --- | --- | --- |
| **TITLE** | | |  |
| Title | 1 | This study is identified as a meta-analysis | 1 |
| **ABSTRACT** | | |  |
| Abstract | 2 | This summary includes the Background, methods, results and discussion | 2 |
| **INTRODUCTION** | | |  |
| Rationale | 3 | Described in the introduction | 2-3 |
| Objectives | 4 | Stated in the introduction | 2-3 |
| **METHODS** | | |  |
| Eligibility criteria | 5 | This paper provided a detailed description of the inclusion and exclusion criteria | 4 |
| Information sources | 6 | Specify all databases, registers, websites, organisations, reference lists and other sources searched or consulted to identify studies. Specify the date when each source was last searched or consulted. | 3-4 |
| Search strategy | 7 | Present the full search strategies for all databases, registers and websites, including any filters and limits used. | 3-4 |
| Selection process | 8 | The screening process is included in the data collection | 5 |
| Data collection process | 9 | We describe the data collection in detail | 5 |
| Data items | 10a | We provide a detailed description of the outcome indicators | 4 |
|  | 10b | List and define all other variables for which data were sought (e.g. participant and intervention characteristics, funding sources). Describe any assumptions made about any missing or unclear information. | 4 |
| Study risk of bias assessment | 11 | We provide a detailed description of the risk assessment tool | 5 |
| Effect measures | 12 | In the methodology section, we made a specific introduction to the measurement of each outcome indicator | 5 |
| Synthesis methods | 13a | Describe the processes used to decide which studies were eligible for each synthesis (e.g. tabulating the study intervention characteristics and comparing against the planned groups for each synthesis (item #5)). | 5-6 |
|  | 13b | Describe any methods required to prepare the data for presentation or synthesis, such as handling of missing summary statistics, or data conversions. | 5-6 |
|  | 13c | Describe any methods used to tabulate or visually display results of individual studies and syntheses. | 5-6 |
|  | 13d | Describe any methods used to synthesize results and provide a rationale for the choice(s). If meta-analysis was performed, describe the model(s), method(s) to identify the presence and extent of statistical heterogeneity, and software package(s) used. | 5-6 |
|  | 13e | Describe any methods used to explore possible causes of heterogeneity among study results (e.g. subgroup analysis, meta-regression). | 5-6 |
|  | 13f | Describe any sensitivity analyses conducted to assess robustness of the synthesized results. | 5-6 |
| Reporting bias assessment | 14 | We introduced the method of measuring bias. | 5-6 |
| Certainty assessment | 15 | We used GRADE to evaluate the evidence for an outcome. | 5 |
| **RESULTS** | | |  |
| Study selection | 16a | Describe the results of the search and selection process, from the number of records identified in the search to the number of studies included in the review, ideally using a flow diagram. | 6 |
|  | 16b | Cite studies that might appear to meet the inclusion criteria, but which were excluded, and explain why they were excluded. | 6 |
| Study characteristics | 17 | Cite each included study and present its characteristics. | 6 |
| Risk of bias in studies | 18 | Present assessments of risk of bias for each included study. | 6-7 |
| Results of individual studies | 19 | For all outcomes, present, for each study: (a) summary statistics for each group (where appropriate) and (b) an effect estimate and its precision (e.g. confidence/credible interval), ideally using structured tables or plots. | 7-10 |
| Results of syntheses | 20a | For each synthesis, briefly summarise the characteristics and risk of bias among contributing studies. | 7-10 |
|  | 20b | Present results of all statistical syntheses conducted. If meta-analysis was done, present for each the summary estimate and its precision (e.g. confidence/credible interval) and measures of statistical heterogeneity. If comparing groups, describe the direction of the effect. | 7-10 |
|  | 20c | We conducted subgroup analysis to further study | 9-10 |
|  | 20d | Present results of all sensitivity analyses conducted to assess the robustness of the synthesized results. | 10 |
| Reporting biases | 21 | Present assessments of risk of bias due to missing results (arising from reporting biases) for each synthesis assessed. | 10 |
| Certainty of evidence | 22 | We used GRADE to evaluate the evidence for an outcome. | 10-11 |
| **DISCUSSION** | | |  |
| Discussion | 23a | Provide a general interpretation of the results in the context of other evidence. | 11-12 |
|  | 23b | Discuss any limitations of the evidence included in the review. | 13 |
|  | 23c | Discuss any limitations of the review processes used. | 13 |
|  | 23d | Discuss implications of the results for practice, policy, and future research. | 11-12 |
| **OTHER INFORMATION** | | |  |
| Registration and protocol | 24a | The registration information is the same as that in this document. Registration No.: CRD42022375440 | 3 |
|  | 24b | Indicate where the review protocol can be accessed, or state that a protocol was not prepared. | / |
|  | 24c | The registration information can be seen on prospero | 3 |
| Support | 25 | Describe sources of financial or non-financial support for the review, and the role of the funders or sponsors in the review. | 13 |
| Competing interests | 26 | No conflict of interest between authors | 13 |
| Availability of data, code and other materials | 27 | All data are published in the public database | 13 |
